# Sleeplessness from Thought

**Published:** 1881-05

**Authors:** 


### SLEEPLESSNESS FROM THOUGHT.

The loss of power to cast off the burden of the day, and find rest in unconsciousness or forgetfulness at night, is one of the greatest of personal afflictions. Only those who have endured it know how terrible this experience, in its worst form, may prove. There is no escape anywhere, no respite, no—even momentary—lessening of the strain on the mind, when sleep is impossible; and the worry is increased when the mind, instead of finding ease, falls into a state in which every source of disquietude seems exaggerated. Sleeplessness of this sort is often the prelude—and it may be either the first indication, or itself the cause—of insanity. The condition into which the mind is thrown when endeavoring to sleep, is essentially unsound, and tends to disease.

Thoughts, passing through the mind when the brain is falling into a state of sleep, ought to be of a nature to change easily into a dream. The problem is to carry the mind over the boundary line, and convert what is conscious but uncontrollable thought into a dream. If this can be accomplished naturally—that is, without the aid of drugs, which stupify the consciousness and burlesque the state of sleep rather than produce it—the subject of thought will be soon changed, and oblivion, or at least forgetfulness, induced. The solution of this problem may be attempted by either of two processes:

1. A particular thought, or train of thoughts, present to the mind may be seized upon at the moment of their occurrence, while as yet they are manageable, and turned into grotesque, thus preparing them to become the material or centre of an amusing dream. This method is less easy to describe than to carry out, but experience proves that it is abundantly efficacious. Fancy must be directed to play with the thought and weave a little scene or story out of its slenderest threads. Just enough effort to preserve the connection of ideas is necessary or the expedient will fail, thought reverting to its former worrying courses. The secret of the method lies in holding the thought fixed, and projecting the train of ideas by fancy on a line which may carry it into dreamland, the dreaminess of thought inducing sleep. This is a perfectly natural and rational process, and it is harmless, whereas the production of stupefaction by drugs is artificial, and more or less perilous to brain and mind. The one lulls the consciousness to sleep; the other overpowers it with a poison.

2. The alternative mental method by which sleep may be sought consists in giving thought a monotonous task in the way suggested by those who can win sleep by counting, repeating, and the like expedients. This is more difficult in really bad cases of "sleeplessness from thought" than that first described, in which an idea or train of ideas, already present to the mind, is converted into grotesque. The mind is not easily taken out of itself when engrossed with worrying topics, and, though fancying corn-fields and rising tides, or counting, and piling up packages, or smoking an imaginary pipe and watching the clouds of tobacco smoke rise over the head, so as to direct the eyes upward as in sleep, are good enough devices, it is not always practicable to shut out distressing or plaguing ideas, and concentrate the attention on these meaningless conceptions for the full success of which the sleep-wooer needs a vacant rather than a harassed mind. It is an effort quite as great as the wakeful, but worried, can make to turn a troublesome thought into grotesque imagery; but this is easier than to call up a wholly new and incongruous idea.

It is a common mistake to plan the business of the following day at night. This is like turning over a new page when the book should be closed and laid aside. The task for laying out schemes for the future ought to be the first duty on waking, and if it were then discharged many mischevious dreams and much of the feeling that a whole night has been spent in dreaming would be avoided. Each night should see the book of life closed, with the feeling that the account has been duly made up. It is the task of the morning to carry over the debt or credit and start afresh. Better by far finish the work of the day, close the record, and seek rest. When the conciousness returns examine the situation, lay plans for the future, and while the impression lasts act on it.

Sleeping and waking are states which are mutually dependent, and must succeed each other in orderly sequence if health is to be preserved. Life is very much an affair of rhymth, and a sound mind can be secured only by concord, method, and orderly self-control by the will.—*Granville* "*Common Mind Troubles.*"

